# Comparative Genomics of the Genus *Methanohalophilus*, Including a Newly Isolated Strain From Kebrit Deep in the Red Sea

**DOI:** 10.3389/fmicb.2019.00839

**Published:** 2019-04-24

**Authors:** Yue Guan, David K. Ngugi, Manikandan Vinu, Jochen Blom, Intikhab Alam, Sylvain Guillot, James G. Ferry, Ulrich Stingl

**Affiliations:** ^1^Red Sea Research Center, King Abdullah University of Science and Technology, Thuwal, Saudi Arabia; ^2^Bioinformatik und Systembiologie, Justus-Liebig-Universität Giessen, Giessen, Germany; ^3^Computational Bioscience Research Center, King Abdullah University of Science and Technology, Thuwal, Saudi Arabia; ^4^Department of Biochemistry and Molecular Biology, The Pennsylvania State University, University Park, PA, United States; ^5^Department of Microbiology and Cell Science, UF/IFAS Fort Lauderdale Research and Education Center, University of Florida, Davie, FL, United States

**Keywords:** deep-sea brine, red sea, methanogenic archaea, *Methanohalophilus*, comparative genomics

## Abstract

Halophilic methanogens play an important role in the carbon cycle in hypersaline environments, but are under-represented in culture collections. In this study, we describe a novel *Methanohalophilus* strain that was isolated from the sulfide-rich brine-seawater interface of Kebrit Deep in the Red Sea. Based on physiological and phylogenomic features, strain RSK, which is the first methanogenic archaeon to be isolated from a deep hypersaline anoxic brine lake of the Red Sea, represents a novel species of this genus. In order to compare the genetic traits underpinning the adaptations of this genus in diverse hypersaline environments, we sequenced the genome of strain RSK and compared it with genomes of previously isolated and well characterized species in this genus (*Methanohalophilus mahii*, *Methanohalophilus halophilus*, *Methanohalophilus portucalensis*, and *Methanohalophilus euhalobius*). These analyses revealed a highly conserved genomic core of greater than 93% of annotated genes (1490 genes) containing pathways for methylotrophic methanogenesis, osmoprotection through salt-out strategy, and oxidative stress response, among others. Despite the high degree of genomic conservation, species-specific differences in sulfur and glycogen metabolisms, viral resistance, amino acid, and peptide uptake machineries were also evident. Thus, while *Methanohalophilus* species are found in diverse extreme environments, each genotype also possesses adaptive traits that are likely relevant in their respective hypersaline habitats.

## Introduction

Hydrogenotrophic, methylotrophic and acetoclastic pathways are the major pathways for methanogenesis – the production of methane by microbial cells (Ferry and Kastead, 2007). Methanogenic species thriving in hypersaline environments have only been reported from the order *Methanosarcinales* and the recently described “Methanonatronarchaeia” (Sorokin et al., 2017) that branch deeply within the Euryarchaeota. Halophilic methylotrophic methanogens belonging to the order *Methanosarcinales* use the classical methylotrophic pathway where methylated compounds are dismutated to methane and CO_2_, whereas “Methanonatronarchaeia” use a heterotrophic methylotrophic pathway in which methylated compounds are used as electron acceptors and formate or hydrogen are being used as electron donors (Sorokin et al., 2017).

In hypersaline environments, the salinity and additional factors such as redox potential, permanency of anaerobic conditions, and the concentrations of other terminal electron acceptors determine the distribution and energy-efficiency of the prevailing methanogenic pathways. In these habitats, methylotrophic methanogens are more successful compared to hydrogenotrophic and acetoclastic methanogens due to their higher energy yield and the use of non-competitive substrates, such as methylamines and methanol (McGenity, 2010; Lazar et al., 2011; Oren, 2011; Kelley et al., 2012). In order to keep the cell cytoplasm iso-osmotic at elevated salt concentrations, both organic osmolytes (“salt-out” strategy) as well as the accumulation of potassium ion (“salt-in” strategy) are used for osmotic balance in *Methanosarcinales* (Lai and Gunsalus, 1992; Sowers and Gunsalus, 1995), while accumulation of potassium alone (“salt-in” strategy) is used to achieve an osmotic equilibrium in the “Methanonatronarchaeia” (Sorokin et al., 2017).

The majority of cultivated halophilic methylotrophic methanogens within the order *Methanosarcinales* belong to the genus *Methanohalophilus*, which currently comprises five described species: *Methanohalophilus halophilus* isolated from Shark Bay in Australia (Zhilina, 1983)*, Methanohalophilus euhalobius* (Obraztsova et al., 1987), *Methanohalophilus mahii* isolated from the Great Salt Lake in Utah, United States (Paterek and Smith, 1988), *Methanohalophilus portucalensis* isolated from a salt pan in Portugal (Boone et al., 1993), and *Methanohalophilus levihalophilus* isolated from a natural gas-bearing deep aquifer (Katayama et al., 2014). The methanogens of the genus *Methanohalophilus* are unique among the halophilic methanogens with respect to their ability to grow in a wide range of salinities (1.5–20% NaCl). All known members of this genus are mesophilic, neutrophilic, obligate methylotrophic methanogens, except for the recently described *M. levihalophilus*, which appears to be only slightly halophilic, growing optimally at NaCl concentration around 2%.

Deep-sea hypersaline anoxic basins (DHABs) are polyextreme brines that so far have been found only on the seafloors of the Mediterranean Sea, the Gulf of Mexico, and the Red Sea. Biologically produced methane is an important metabolite in the DHABs (Borin et al., 2009) and marker gene sequences that are closely related to those of *Methanohalophilus* have been commonly found in deep-sea hypersaline environments (La Cono et al., 2011; Yakimov et al., 2013; Guan et al., 2015). One methanogenic strain, *Methanohalophilus* sp. TA21, has been isolated from Lake Thetis in the Eastern Mediterranean Sea (Cono et al., 2015). This strain performs methanogenesis from methylotrophic substrates arising from the catabolism of glycine betaine by other microbial consortia in the brine.

In several deep-sea brine habitats of the Red Sea, *Methanosarcinales* have been identified as the main methanogens based on genetic markers (Guan et al., 2015), but our knowledge of *Methanohalophilus* in the Red Sea is limited to the presence, distribution and relative abundance of marker gene sequences (Guan et al., 2015). In this study, we describe the isolation and the physiological characteristics of the first strain affiliated with this genus from the brine-sea water interface of Kebrit Deep together with its genome sequence to obtain a better understanding of its metabolic potential. Additionally, we describe comparative genome analyses with multiple, closely related *Methanohalophilus* species, providing insights into their life styles and adaptive strategies to different harsh environments.

## Materials and Methods

### Sample Collection

The samples of the brine-seawater interface (BSI) and the brine were collected by Dr. André Antunes from of the Kebrit Deep (24° 43′ 28″N, 36° 16′ 36″E) by means of a rosette sampler at depths of 1,467 and 1,490 m below the sea surface during the R/V Aegaeo Red Sea Expedition in November 2011. At the time of sampling, the methane concentration was 1293 μM and temperatures ranged between 21.9 and 23.4°C, while salinity levels were between 18.2 and 26% (Guan et al., 2015; Ngugi et al., 2015).

### Enrichments and Isolation

Enrichments were initiated by adding 5 ml of Kebrit Deep samples to anaerobic serum vials that contained 45 ml of medium containing 50 μM trimethylamine (TMA) and a bicarbonate buffer system previously used to isolate marine methanogens (Sowers and Ferry, 1983; Sowers et al., 1984). The cultures were incubated in the dark at either 22, 30, or 37°C. Enrichment cultures were screened after approximately 4 weeks of incubation for methane production (using Agilent 6890 Gas Chromatography/Mass Spectrometry, Agilent, Santa Clara, CA, United States) and increase in turbidity (measuring optical density at 600 nm, SpectraMax, Molecular Devices, San Jose, CA, United States), and, if positive, further isolation of single colonies on agar plates (using the same medium, incubated under anoxic conditions) resulted in the isolation of strain RSK. The purity of the culture was tested by ensuring cells of uniform morphology with light microscopy as well as sequencing of 16S rRNA genes, and finally confirmed by the analyses of the genome sequences of the cultures.

### Characterization

The effect of different salinity ranges on growth of strain RSK was tested in the above-mentioned enrichment media containing NaCl in 1% increments ranging from 0 to 30%. Tests were performed in triplicate in Hungate anaerobic culture tubes as described previously (Sowers et al., 1984; L’Haridon et al., 2014), and growth was evaluated by measuring absorbance of the cultures at 600 nm. The range of temperatures supporting growth and the optimal growth temperature of strain RSK was tested at 4, 15, 22, 30, 33, 35, 37, 40, 45, and 50°C. The pH range for growth was examined between pH 5 and pH 9 with increments of 0.5. Formate (50 mM), acetate (90 mM), methanol (150 mM), H_2_:CO_2_ (4:1 v/v), and methylated substrates (mono-, di-, and tri-methylamine at 50 mM each, betaine at 5 mM each) were tested as potential growth substrates. The effects of the addition of vitamins biotin, folic acid, vitamin B_12_, pyridoxine-HCl, thiamine-HCl, riboflavin, nicotinic acid, pantothenate, *p*-aminobenzoic acid, lipoic acid with concentrations used previously (Sowers et al., 1984; Wolfe, 2011), and stimulatory compounds (e.g., yeast extract, peptone tested in three final concentrations 0.05, 1, and 2 g/L) on growth parameters of strain RSK were also tested. Inhibition of growth by antibiotics (ampicillin, kanamycin, carbenicillin, penicillin G, chloramphenicol, rifampicin, and vancomycin at final concentrations of 100 μg per ml) was examined with 50 μM TMA as the substrate.

### Microscopy

For imaging using Transmission Electron Microscopy (TEM), cells in exponential phase growing with TMA as substrate were fixed by adding 2.5% glutaraldehyde directly to the growth medium. Transmission electron micrographs were obtained in the Imaging and Characterization Core Lab at King Abdullah University of Science and Technology (KAUST) using a Titan G2 80–300 kV TEM (FEI). The TEM was equipped with a 4 k × 4 k CCD camera (US4000) and an energy filter, model GIF Tridiem (Gatan, Inc., Germany). Cyro-scanning electron microscopy was performed using a PP2000T cryo-transfer system (Qurorum Technologies) attached to an FEI Nova Nano630 scanning electron microscope equipped with a field emission electron source and through-the-lens electron detectors.

### Whole-Genome Sequencing, Assembly, Annotation, and Comparative Genomics

Genomic DNA of strain RSK grown with TMA was extracted and purified using the DNeasy Blood & Tissue Kit (Qiagen, Hilden, Germany) following the manufacturer’s protocols. Genomic DNA of *M. euhalobius* (DSM 10369), *M. halophilus* (DSM 3094), and *M. portucalensis* (DSM 7471) was provided by DSMZ (German Collection of Microorganisms and Cell Cultures). Sequencing libraries were prepared with 1 μg genomic DNA using the Illumina TruSeq^TM^ DNA sample preparation kit. Sequencing was done using an Illumina MiSeq sequencer at KAUST’s Bioscience Core Laboratory following Illumina’s protocols. Reads were quality filtered, trimmed, and assembled into contigs using the *de novo* assembler SPAdes, version 2.5.1 (Bankevich et al., 2012).

Putative coding sequences (CDSs) of all draft genomes were predicted using the automated annotation pipeline INDIGO^1^ (Alam et al., 2013). Clustered Regularly Interspaced Short Palindromic Repeats (CRISPR) loci, CRISPR direct repeat elements (DR), and spacers were detected by CRISPRFinder online server (Grissa et al., 2007). Small CRISPRs (e.g., CRISPR loci with less than three DRs) were marked as questionable CRISPRs. CRISPRmap (Lange et al., 2013) was used to classify the class assignment and structure motifs of DRs.

The relatedness of the organisms at the genome level was assessed using the average nucleotide identity (ANI) metric and the overall gene order conservation (synteny) as described in Ngugi et al. (2015). The complete genome of *M. mahii* (Spring et al., 2010) was used as a reference for the comparative analysis. To synchronize the genome annotation, the published genome of *M. mahii* was re-annotated using the INDIGO pipeline (Alam et al., 2013). Comparative genomics was conducted using the phylogenomic pipeline EDGAR (Blom et al., 2009), as described in Ngugi et al. (2015).

Publicly available genomes representing all methanogenic archaea as well as a few *Thermoplasmata* species were selected for a comprehensive phylogenetic analysis of the genus *Methanohalophilus* and downloaded from GenBank^2^. Subsequently, AMPHORA2 (Wu and Scott, 2012) was used to identify a total of 77 single conserved marker genes for phylogenomic reconstruction. The phylogeny was inferred by a maximum likelihood approach with Le Gascuel’s substitution model under 100 bootstrap replicates implemented in PHYML as implemented in Geneious R9 Biomatters, Ltd.; (Kearse et al., 2012).

### Data Submission

The draft genome sequences of strain RSK, *M. portucalensis*, *M. halophilus*, and *M. euhalobius* are deposited in Genbank under BioProject identifier PRJNA500754. These genome drafts of *M. portucalensis* and *M. halophilus* presented here were used in the publications by L’Haridon et al. (2017, 2018).

## Results and Discussion

### Isolation and Physiological Characteristics of Strain RSK

At the time of sampling, remarkably high concentrations of methane (1293 μM), H_2_S (149.8 μM), and CO_2_ (4630 μM) were observed in Kebrit Deep BSI relative to other deep-sea brines of the Red Sea (Guan et al., 2015). Marker gene profiling suggested that sulfate-reducing bacteria and methylotrophic methanogenic *Methanohalophilus* populations co-inhabit this location, possibly because they do not compete for energy substrates (Lovley et al., 1982; Oremland and Polcin, 1982; McGenity, 2010; Guan et al., 2015). Strain RSK was isolated from an enrichment culture inoculated with brine-seawater interface samples from Kebrit Deep (1,468 meters below the sea surface) in the Red Sea. This strain represents the first methanogenic culture from the deep-sea brines of the Red Sea.

Strain RSK utilizes mono-, di-, and trimethylamine as well as methanol as energy sources, but no growth was observed when hydrogen (head space H_2_:CO_2_, 4:1 v/v), formate or acetate were used as substrates. Strain RSK was able to grow in a broad salinity range (2–20% NaCl), which is the widest among all described *Methanohalophilus* species so far (Table 1). Methane was still produced at 25% NaCl although growth was not observed. The temperature range for growth was 15–40°C. No growth was observed at 45°C and above over a period of 6 months. Optimal growth was observed in a medium containing 5 to 10% NaCl, 33°C and a pH of 6.5. Vitamins, yeast extract and trypticase did not stimulate growth. RSK was resistant to ampicillin, kanamycin, carbenicillin, penicillin G, and vancomycin, while chloramphenicol and rifampicin inhibited its growth (as determined by OD_600_, Table 1).

**Table 1:** Characteristics of strain RSK and closely related species in the genus *Methanohalophilus*.

Characteristics	*Methanohalophilus* strain RSK	*Methanohalophilus mahii* (DSM 5219)	*Methanohalophilus halophilus* (DSM 3094)	*Methanohalophilus portucalensis* (DSM 7471)	*Methanohalophilus euhalobius* (DSM 10369)
Main publication	This study	Paterek and Smith (1988)	Zhilina (1983)	Boone et al. (1993)	Obraztsova et al. (1987)
cell shape	cocci	irregular cocci	irregular cocci	irregular cocci	irregular cocci
Isolation source	Kebrit BSI, Red Sea	Great Salt Lake, Utah, sediment	Shark Bay, Australia	Salinarium in Figiera da Foz, Portugal	Saline subsurface water, Russia, Bonduzhkoe oil deposit
Size (μm)	0.3–2.5	0.8–1.8	Not reported	0.8–1.2	
Motility	non-motile	non-motile	non-motile	non-motile	non-motile
Temp. range (°C)	15–40 (no growth at 4 or 45)	< 45	18 – 42^1^	30 – 42	15 – 50
Temp. optima (°C)	30 – 33	35 – 37	26 – 36	37 – 42	28 – 37
pH range	5.5–7.5	6.5–8.5	6.5–7.4	6.0–8.5	5.8–8.0
Optimal pH	6.5	7.5	7.4	7.2	6.8–7.3
NaCl range (%)	2–20	2.9–14.6	1.5–15^1^	8.2–17.5^2^	1–14
Optimal NaCl (%)	5–10	11.7	7–9	12.9	6
**Substrates**					
TMA	+	+	+	+	+
DMA	+	+	+	+	+
MMA	+	+	+	+	+
Methanol	+	+	+	+	+
Glycine betaine	−	Not reported	Not reported	Not reported	Not reported
Acetate	−	−	−	−	−
Formate	−	−	−	−	−
H_2_/CO_2_	−	−	−	−	−
Growth factors	not required	not reported	not required	biotin	biotin

*^1^The temperature range was 18–40°C and salinity range was 1.75–15.2% according to Wilharm et al., 1991. ^2^The salinity range was 2.9–20.5% for a number of M. portucalensis strains described by Boone et al. (1993).*

### Cell Morphology

**FIGURE 1:**
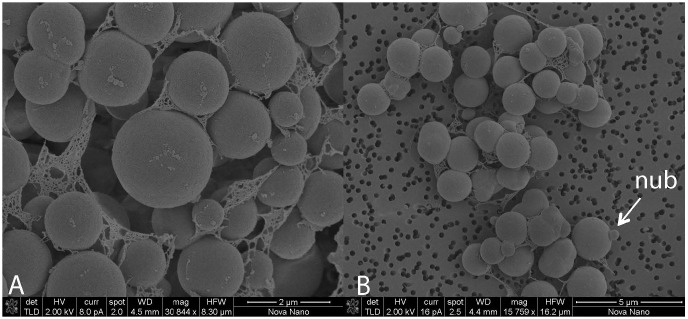
Transmission electron microscopy (TEM) micrographs of cell aggregates of strain RSK. **(A)** Magnification 30844 x, **(B)** Magnification 15759 x.

RSK cells growing with trimethylamine were non-motile, mostly regular cocci ranging from 0.3 to 2.5 μm in diameter. Single cells, pairs, or small aggregates were detected. Small nubs on the cell surface were observed, which could be extracellular vesicles or buddings due to their sizes and bud-like morphological characteristics (Choi et al., 2015; Figure 1). Compared to previously described *Methanohalophilus* species (Spring et al., 2010; Katayama et al., 2014), regular cocci-shaped cells and nubs are two distinct cellular features of strain RSK that have not been reported from other strains.

### Comparative Genomics of *Methanohalophilus* Species

**FIGURE 2:**
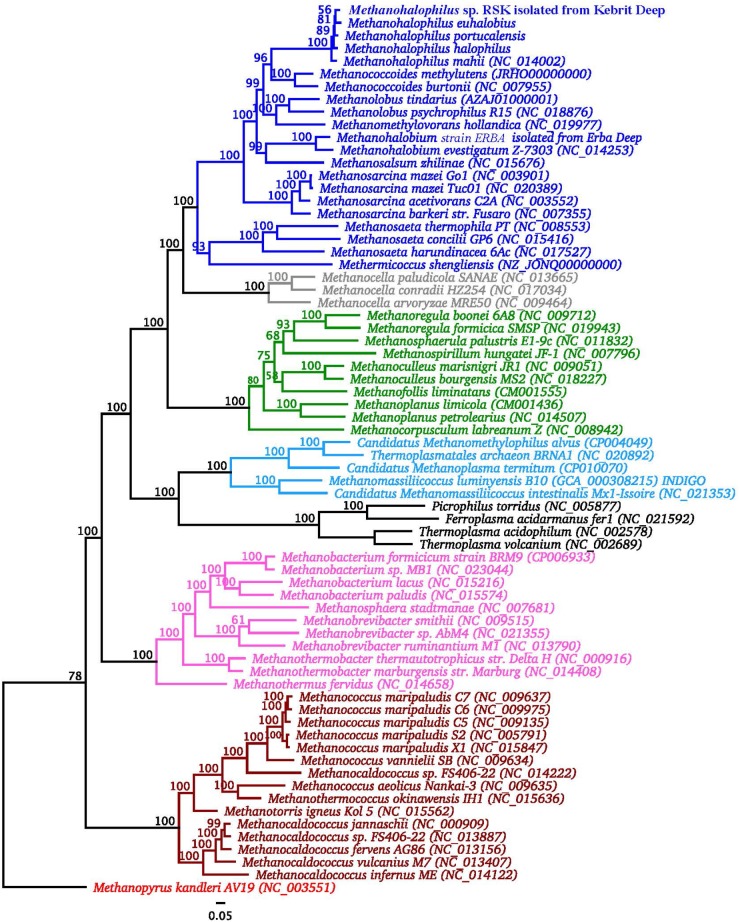
Phylogenomic analysis of strain RSK in relation to other *Methanohalophilus* spp. and other methanogens. The tree is rooted with sequences of *Methanopyrus kanderi*. Significance of branch support inferred using maximum likelihood (*n* = 100 bootstraps). Strain RSK is in bold. *Methanosarcinales* are shown in blue, *Methanocellales* are shown in gray, *Methanomicrobiales* are shown in green, *Methanomassiliicoccales* are shown in light blue, *Methanobacteriales* are shown in purple, *Methanococcales* are shown in ochre.

An overview of genomic characteristics of five *Methanohalophilus* species studied here is summarized in Table 2. Based on one complete genome and four high-quality draft genomes with >99.5% estimated genome completeness, the average genome length of *Methanohalophilus* species is around 2.0 Mbp, making them the smallest genomes in the family *Methanosarcinaceae* [ranging from around 2.0 to 5.75 Mbp (Galagan et al., 2002)]. The DNA G+C content of the genomes of *Methanohalophilus* were almost identical at around 42%, while the numbers of predicted open-reading frames ranged from 1,990 to 2,124. All tRNAs for 20 common proteinogenic amino acids and pyrrolysine are present. Species of this genus are also very similar to each other at the 16S rRNA gene level (>99.4%), in their tetranucleotide signatures (>0.99%), and based on their genomic average nucleotide identities (ANI; 91.1–93.7%; Supplementary Table S1). Thus, based on an ANI threshold of ∼95%, which is currently being operationally used for species delineation (Konstantinidis and Tiedje, 2005), our results indicate that strain RSK represents a novel species in the genus *Methanohalophilus*. *M. euhalobius* is the most closely related species, based on phylogenomic analysis of 77 single conserved marker genes (Figure 2).

**Table 2:** Assembly statistics and genome features of sequenced *Methanohalophilus* species.

Species	*M. mahii*	*M. halophilus*	*M. portucalensis*	*M. eubalobius*	Strain RSK
Reference	Spring et al., 2010	This study^2^	This study^2^	This study	This study
Size (bp)	2012424	2021331	2080338	1875790	1969036
Completeness (%)^1^	99.35	99.51	99.51	99.51	99.51
No. of contigs	1	6	17	25	18
ORFs	2016	2047	2124	1990	2053
DNA G+C content (%)	42.6	42.4	41.9	42.4	41.87
rRNA operon (16S-23S-5S)	3	1	1	1	1
Unique proteins within genus *Methanohalophilus*	131 (6.50%)	151 (7.38%)	216 (10.17%)	276 (13.39%)	260 (12.7%)
CRISPR repeats	0	0	1	2	3

*^1^Estimated using 228 marker genes in Euryarchaeota genomes using Check M (Parks et al., 2015). ^2^These genome drafts of M. portucalensis and M. halophilus presented here were used in the publications by L’Haridon et al. (2017, 2018) with Genbank accession numbers CP017881 and CP017921, respectively.*

**FIGURE 3:**
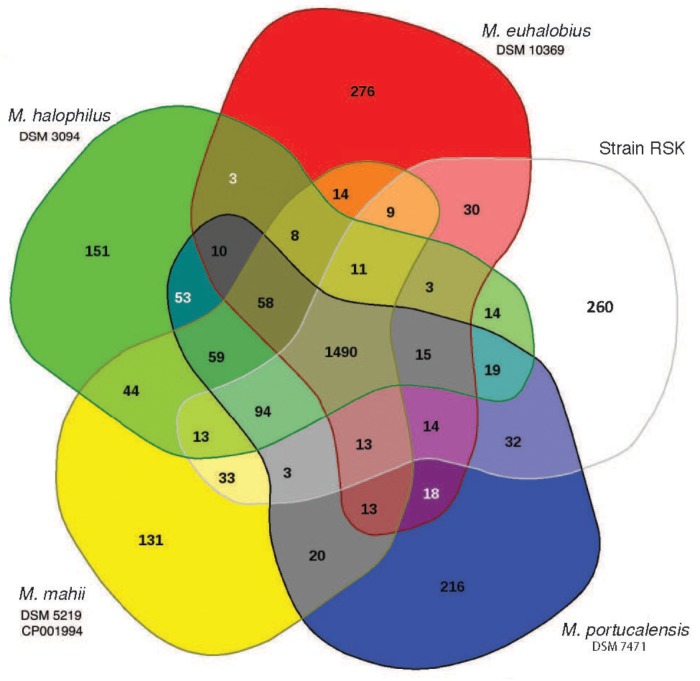
Comparative analysis of the predicted proteomes of *Methanohalophilus* species analyzed in EDGAR. The Venn diagram shows the core, unique, and flexible genome components among strains RSK (Red Sea Kebrit BSI isolate), *M. mahii*, *M. portucalensis*, *M. euhalobius*, and *M. halophilus*.

Complementary synteny-based comparative genomics analysis (Yelton et al., 2011) comparing the predicted proteome of strain RSK with that obtained from the completed genome of *M. mahii* shows that ∼77% of their core genes are syntenic, implying a high degree of functional conservation between these two species. We estimate that the core genome of strain RSK and closely related species of *Methanohalophilus* contains 1,490 protein-coding genes, accounting for 70 to 75% of their predicted proteomes (Figure 3), which is significantly larger than the core genome formed by including representatives of *M. euhalobius* recovered from hydraulically fractured rocks in unconventional reservoirs (Borton et al., 2018). Only 6.5–13.4% of all predicted proteins are unique to the five individual species compared in this study.

### Core Metabolic Features of *Methanohalophilus* Species

The genome of *Methanohalophilus* strain RSK is the first genome of methanogen from DHABs. It shares very similar metabolic capacities with closely related of this genus with respect to energy metabolism and strategies for oxidative and osmotic stress response. These genomes encode the complete set of enzymes for methanogenesis from methyl compounds but lack the hydrogenotrophy-associated hydrogenases, acetate kinase and acetate thiokinase encoding genes necessary growth with H_2_-CO_2_ and acetate, respectively (Ferry, 2010).

#### Oxidative Adaptation

Methanogens inhabit a diverse array of ecosystems with a broad range of physicochemical conditions, including some habitats where oxygen is constantly present at low concentrations or is present in measurable concentrations at certain times, e.g., the BSIs of the deep-sea brines of the Red Sea and soil crusts (Antunes et al., 2011; Guan et al., 2015; Ngugi et al., 2015). However, high oxygen concentrations are usually restrictive to their anaerobic lifestyle and thus their distribution (Hoehler et al., 2010). Interestingly, *Methanohalophilus* species are commonly found in brine-seawater transition zones (La Cono et al., 2011; Yakimov et al., 2013, 2014; Guan et al., 2015), where micro-oxic conditions exist (Ngugi et al., 2015). Strain RSK was isolated from the BSI of Kebrit Deep, where the concentration of dissolved oxygen (DO_2_) was 4.6 μM. Thus, it must have developed mechanisms to protect themselves from the toxicity of DO_2_ and reactive oxygen species (ROS) and to repair damaged cellular compounds and enzymes. Accordingly, the genomes of strain RSK and type strains of *Methanohalophilus* encode up to eight copies of thioredoxin, up to six copies of different isozymes of catalase peroxidase, peroxiredoxin, alkyl-hydroperoxide reductase, and alkyl-hydroperoxidase, and three copies of rubrerythrin. Thioredoxin reductase, catalase, and superoxide reductase were also detected in each genome. Among the above mentioned enzymes encoded by *Methanohalophilus* genomes, thioredoxin reductase and thioredoxin together form the thioredoxin system which constitutes a key function in defense against oxidative stress in providing thioredoxin-based oxidative redox regulation (Lu and Holmgren, 2014; Susanti et al., 2014). The *Methanohalophilus* genomes encode alkyl-hydroperoxide reductase, peroxiredoxin, catalase, catalase peroxidase that could reduce hydrogen peroxide (Seaver and Imlay, 2001; Sakai et al., 2011), alkyl-hydroperoxidase and rubrerythrin, which could reduce peroxides (Sztukowska et al., 2002; Guimarães et al., 2005), and superoxide reductase, which could detoxify superoxide.

Glutaredoxins are small ubiquitous thiol-disulfide redox enzymes (Iversen et al., 2010). In the domain *Bacteria*, glutaredoxin is reduced by glutathione and diverse low-molecular-weight thiols (Holmgren, 1989; Van Laer et al., 2013). Prevailing evidence indicates that glutathione and glutaredoxins are unlikely participants in the oxidative stress response of methanogens (McCarver et al., 2017; Prakash et al., 2018). In *Methanosarcina acetivorans*, the annotated glutaredoxin enzyme (AAM05066.1, renamed methanoredoxin) utilizes coenzyme M and ferredoxin:disulfide reductase (Yenugudhati et al., 2015; Prakash et al., 2018). A glutaredoxin homolog, which is more similar to glutaredoxin from the domain *Bacteria* (30% identity, 71% coverage to glutaredoxin under the accession number Q9HU55.1 from *Pseudomonas aeruginosa* PAO1), is also present in each genome of *Methanohalophilus* reported here. Further biochemical characterization of this enzyme from *Methanohalophilus* would be necessary to identify its actual function.

#### Osmotic Adaptation

Osmotic adaptation is one of the main factors that allow halophilic methanogens to thrive in hypersaline environments. All *Methanohalophilus* species compared in this study have the genomic capacity to perform both the “salt-in” and “compatible solute” strategies (Oren, 2011) to combat osmotic stress as described for *M. mahii* (Spring et al., 2010). The presence of a K^+^/Na^+^ symporter Ktr as well as multiple copies of the potassium uptake system Trk and the glycine betaine/L-proline transport system permease (ProW) in all analyzed genomes of *Methanohalophilus* indicate that the “salt-in” strategy is widely used by these methanogens to cope with salinity. Notably, all strains possess the potential to synthesize betaine (unique to this genus and *Methanohalobium*) by methylation of glycine (Chen et al., 2009; Lai and Lai, 2011; Borton et al., 2018) and to synthesize *N*^ε^-acetyl-β-lysine from lysine; the later trait was also detected in the genome of closely related halophilic methanogens of genus *Methanococcoides* (Guan et al., 2014) and several other methanogens (Schlegel and Müller, 2011).

### Flexible Gene Sets of *Methanohalophilus* Species

#### Sulfur Metabolism

Sulfite is toxic to methanogens as it inactivates the essential enzyme for methanogenesis, methyl-coenzyme M reductase (Mahlert et al., 2002). In the brine-seawater interface of Kebrit Deep, where strain RSK was isolated from, sulfite is likely generated due to the presence of oxygen (4.6 μM) and high sulfide concentrations (149.8 μM) (Ngugi et al., 2015). *Methanohalophilus* species have one to two copies of nitrite and sulfite reductase (anaerobic sulfite reductase, MhpRSK_00883) containing a dissimilatory sulfite reductase subunit A domain (DsrA, COG2221), which presumably helps to protect halophilic methanogens in sulfidic environments by reducing sulfite.

*Methanohalophilus portucalensis* it is the only genome predicted to have the complete set of genes to assimilate sulfate catalyzed by sulfate adenylyltransferase (CysD and CysN/C), 3′-phosphoadenosine 5′-phosphosulfate synthase (adenylylsulfate kinase, PAPSS) and phosphoadenosine phosphosulfate reductase, although this would require additional experimental verification. The other four *Methanohalophilus* species lack PAPSS, while sulfate adenylyltransferase was not detected in *M. halophilus* and *M. euhalobius*.

#### ABC Transporters

Members of the genus *Methanohalophilus* appear to have different genomic capacities for the uptake of amino acids and peptides. A predicted operon for peptide transport (Opp) (Goodell and Higgins, 1987) is found only in *M. mahii*, *M. halophilus*, and strain RSK, indicating that they can directly take up peptides as a nutrient source.

The *livK* genes, encoding a high-affinity branched-chain amino acid transport system, are only present in the genomes of *M. mahii*, *M. portucalensis*, and *M. halophilus* (*M. euhalobius* has only a truncated version of this gene). This periplasmic Leu/Ile/Val-binding protein serves as the primary receptor for the leucine transport system in *Escherichia coli* (Anderson and Oxender, 1977; Magnusson et al., 2004). In turn, this suggests variability in the ability of different *Methanohalophilus* members to accumulate exogenous branched-chain amino acids.

#### Glycogen Synthesis and Utilization

Glycogen is a reserve polysaccharide common in methanogens (Simpson and Whitman, 1993). Enzymes for glycogen metabolism (glycogen synthase, alpha-amylase, and glycogen debranching protein) are predicted to be present only in *M. mahii* and *M. halophilus*, suggesting the capacity of these species to synthesize and store glycogen.

#### Viral Defense

As the most abundant biological entities on Earth, viruses are also present in the Deep-sea Anoxic Brines of the Red Sea (Antunes et al., 2015). Clustered regularly interspaced short palindromic repeats (CRISPR)-Cas systems provide prokaryotes with acquired, adaptive, and heritable resistance against foreign invading genetic elements such as viruses, phages, and plasmids (Barrangou et al., 2007; Horvath and Barrangou, 2010). Genes related to viral defense were previously detected in *Methanohalophilus* (Borton et al., 2018). In agreement with this report, we also found that strain RSK possesses complete CRISPR/Cas systems. Genomes of strain RSK and *M. euhalobius* carry two different Cas arrays – Type I and Type III-U, based on the classification proposed by Makarova et al. (2011). Both Type III-U Cas arrays exhibit high sequence similarities and high synteny. Similar Cas arrays could only be found in the genomes of *Candidatus* Methanoperedens nitroreducens and *Methanospirillum hungatei* so far, suggesting a novel variant of subtype III-U. This further indicates the uniqueness of this subtype in anaerobic methane metabolizing microbes. The CRISPR locus 2 in strain RSK and *M. euhalobius* share the same direct repeat element (GTTACCATTCCCTATTTTCTCGGGAGCTACTTTCAAC) (Supplementary Table S2), suggesting close evolutionary relationships in viral defense systems between these two species.

### Unique Genes in Strain RSK Compared to Other Genomes of *Methanohalophilus*

The 260 predicted species-specific genes in the genome of RSK encompass 15 COG categories and are overrepresented in categories “Replication, recombination and repair” (22 genes), “Cell wall/membrane/envelope biogenesis” (17 genes), “Inorganic ion transport and metabolism” (14 genes), “General function prediction only” (14 genes), and “coenzyme transport and metabolism” (12 genes; Supplementary Table S3).

The genome of RSK encodes an aquaporin homolog (RSK_01503) that is 54% identical (coverage 96%) to the aquaporin characterized from *Methanothermobacter marburgensis* str. Marburg (Kozono et al., 2003). This archaeal type of aquaporin showed characteristics of both, aquaporin and aquaglyceroporin, the two existing classes of this protein family (Araya-Secchi et al., 2011). The presence of this gene implies a role for water channel mediated osmotically driven water flux in strain RSK that might aid cells to cope with sudden changes in salinity in their habitat.

The genome of RSK carries putative genes that encode for membrane components of the *ssu* system, which is involved in sulfur assimilation from aliphatic sulfonates during sulfur-starvation conditions. In this system, the *ssuABC* gene products are involved in the import of aliphatic sulfonates while the *ssuDE* gene products form an oxygenase system responsible for releasing sulfur from aliphatic sulfonates (van Der Ploeg et al., 2001). Strain RSK also encodes for a putative aliphatic sulfonate-binding protein SsuA (RSK_00452), a probable aliphatic sulfonate transport permease SsuC (RSK_00453), and an aliphatic sulfonate import ATP-binding protein SsuB (RSK_00454). This suggests that RSK could take up organic sulfur, although the exact mechanism of desulfonation remains unknown.

Furthermore, a partial arsenite efflux protein ArsB that shares 59% amino acid sequence identity to that of *Alkaliphilus metalliredigens* QYMF (acc. A6TP80.1) (Fu et al., 2009) is also encoded in the genome of RSK (RSK_01501). If functional, it would allow RSK to confer resistance to toxic arsenite by pumping it out of the cells.

## Conclusion

Methanogens are important players in the carbon cycle of hypersaline habitats. However, not much is known about the characteristics of methanogens from the deep-sea brine habitats of the Red Sea and no cultures existed. In this study, we describe a novel isolate (strain RSK) from the BSI of Kebrit Deep of the Red Sea representing a novel species within the genus *Methanohalophilus.* In order to understand why species of the genus *Methanohalophilus* are successful in chemically diverse hypersaline environments, we analyzed and compared the genomic inventory among five *Methanohalophilus* species to elucidate genomically conserved traits and underlying mechanisms for oxidative and osmotic adaptation. Their genomic toolboxes encompass versatile metabolic strategies potentially contributing to their osmo-adaptation in polyextreme hypersaline environments. We suggest that niche adaptation of *Methanohalophilus* populations is driven by their different genomic capacities in sulfur and glycogen metabolisms, viral resistance, amino acid and peptide uptake.

## Author Contributions

US conceived and designed the study. YG, DN, and MV, performed the analysis of the sequence data and bioinformatics tasks. JB and IA developed and maintained EDGAR and INDIGO bioinformatic platforms. JF designed the archaeal growth strategy. YG and SG performed the laboratory work. YG and US wrote the manuscript. YG, DN, JF, and US critically reviewed and edited the manuscript. All authors approved the final version of the manuscript.

## Conflict of Interest Statement

The authors declare that the research was conducted in the absence of any commercial or financial relationships that could be construed as a potential conflict of interest.

## Footnotes

1 www.cbrc.kaust.edu.sa/indigo

2 https://www.ncbi.nlm.nih.gov/genbank/

## References

[B1] Alam I. Antunes A. Kamau A. A. Ba alawi W. Kalkatawi M. Stingl U. (2013). INDIGO – integrated data warehouse of MIcrobial GenOmes with examples from the Red Sea extremophiles. *PLoS One* 8:e82210. 10.1371/journal.pone.0082210 24324765 PMC3855842

[B2] Anderson J. J. Oxender D. L. (1977). *Escherichia coli* transport mutants lacking binding protein and other components of the branched-chain amino acid transport systems. *J. Bacteriol.* 130 384–392. 323236 10.1128/jb.130.1.384-392.1977 PMC235216

[B3] Antunes A. Alam I. Simões M. F. Daniels C. Ferreira A. J. S. Siam R. (2015). First Insights into the viral communities of the deep-sea aoxic brines of the Red Sea. *Genom. Proteom. Bioinform.* 13 304–309. 10.1016/j.gpb.2015.06.004 26529193 PMC4678784

[B4] Antunes A. Ngugi D. K. Stingl U. (2011). Microbiology of the Red Sea (and other) deep-sea anoxic brine lakes. *Environ. Microbiol. Rep.* 3 416–433. 10.1111/j.1758-2229.2011.00264.x 23761304

[B5] Araya-Secchi R. Garate J. A. Holmes D. S. Perez-Acle T. (2011). Molecular dynamics study of the archaeal aquaporin AqpM. *BMC Genomics* 12(Suppl. 4):S8. 10.1186/1471-2164-12-S4-S8 22369250 PMC3287591

[B6] Bankevich A. Nurk S. Antipov D. Gurevich A. A. Dvorkin M. Kulikov A. S. (2012). SPAdes: a new genome assembly algorithm and its applications to single-cell sequencing. *J. Comput. Biol.* 19 455–477. 10.1089/cmb.2012.0021 22506599 PMC3342519

[B7] Barrangou R. Fremaux C. Deveau H. Richards M. Boyaval P. Moineau S. (2007). CRISPR provides acquired resistance against viruses in prokaryotes. *Science* 315:1712. 10.1126/science.1138140 17379808

[B8] Blom J. Albaum S. P. Doppmeier D. Pühler A. Vorhölter F.-J. Zakrzewski M. (2009). EDGAR: a software framework for the comparative analysis of prokaryotic genomes. *BMC Bioinformatics* 10:154. 10.1186/1471-2105-10-154 19457249 PMC2696450

[B9] Boone D. R. Mathrani I. M. Liu Y. Menaia J. A. G. F. Mah R. A. Boone J. E. (1993). Isolation and characterization of *Methanohalophilus portucalensis* sp. nov. and DNA reassociation study of the genus *Methanohalophilus*. *Int. J. Syst. Bacteriol.* 43 430–437. 10.1099/00207713-43-3-430

[B10] Borin S. Brusetti L. Mapelli F. D’Auria G. Brusa T. Marzorati M. (2009). Sulfur cycling and methanogenesis primarily drive microbial colonization of the highly sulfidic Urania deep hypersaline basin. *Proc. Natl. Acad. Sci. U.S.A.* 106 9151–9156. 10.1073/pnas.0811984106 19470485 PMC2685740

[B11] Borton M. A. Daly R. A. O’Banion B. Hoyt D. W. Marcus D. N. Welch S. (2018). Comparative genomics and physiology of the genus *Methanohalophilus*, a prevalent methanogen in hydraulically fractured shale. *Environ. Microbiol.* 20 4596–4611. 10.1111/1462-2920.14467 30394652

[B12] Chen S.-Y. Lai M.-C. Lai S.-J. Lee Y.-C. (2009). Characterization of osmolyte betaine synthesizing sarcosine dimethylglycine N-methyltransferase from *Methanohalophilus portucalensis*. *Arch. Microbiol.* 191 735–743. 10.1007/s00203-009-0501-z 19693490

[B13] Choi D. H. Kwon Y. M. Chiura H. X. Yang E. C. Bae S. S. Kang S. G. (2015). Extracellular vesicles of the hyperthermophilic archaeon “*Thermococcus onnurineus*” NA1T. *Appl. Environ. Microbiol.* 81 4591–4599. 10.1128/AEM.00428-15 25934618 PMC4551206

[B14] Cono V. Arcadi E. Spada G. Barreca D. Laganà G. Bellocco E. (2015). A three-component microbial consortium from deep-sea salt-saturated anoxic lake Thetis links anaerobic glycine betaine degradation with methanogenesis. *Microorganisms* 3 500–517. 10.3390/microorganisms3030500 27682102 PMC5023251

[B15] Ferry J. G. (2010). How to make a living by exhaling methane. *Annu. Rev. Microbiol.* 64 453–473. 10.1146/annurev.micro.112408.134051 20528692

[B16] Ferry J. G. Kastead K. A. (2007). “Methanogenesis,” in *Archaea: Molecular and Cellular Biology*, ed. Cavicchioli R. (Washington, DC: ASM Press), 288–314. 10.1128/9781555815516.ch13

[B17] Fu H.-L. Meng Y. Ordóñez E. Villadangos A. F. Bhattacharjee H. Gil J. A. (2009). Properties of arsenite efflux permeases (Acr3) from *Alkaliphilus metalliredigens* and *Corynebacterium glutamicum*. *J. Biol. Chem.* 284 19887–19895. 10.1074/jbc.M109.011882 19494117 PMC2740414

[B18] Galagan J. E. Nusbaum C. Roy A. Endrizzi M. G. Macdonald P. FitzHugh W. (2002). The genome of *M. acetivorans* reveals extensive metabolic and physiological diversity. *Genome Res.* 12 532–542. 10.1101/gr.223902 11932238 PMC187521

[B19] Goodell E. W. Higgins C. F. (1987). Uptake of cell wall peptides by *Salmonella typhimurium* and *Escherichia coli*. *J. Bacteriol.* 169 3861–3865. 10.1128/jb.169.8.3861-3865.1987 3301822 PMC212484

[B20] Grissa I. Vergnaud G. Pourcel C. (2007). CRISPRFinder: a web tool to identify clustered regularly interspaced short palindromic repeats. *Nucleic Acids Res.* 35 W52–W57. 10.1093/nar/gkm360 17537822 PMC1933234

[B21] Guan Y. Hikmawan T. Antunes A. Ngugi D. Stingl U. (2015). Diversity of methanogens and sulfate-reducing bacteria in the interfaces of five deep-sea anoxic brines of the Red Sea. *Res. Microbiol.* 166 688–699. 10.1016/j.resmic.2015.07.002 26192212

[B22] Guan Y. Ngugi D. K. Blom J. Ali S. Ferry J. G. Stingl U. (2014). Draft genome sequence of an obligately methylotrophic methanogen, *Methanococcoides methylutens*, isolated from marine sediment. *Genome Announc.* 2:e1184-14. 10.1128/genomeA.01184-14 25414501 PMC4239356

[B23] Guimarães B. G. Souchon H. Honoré N. Saint-Joanis B. Brosch R. Shepard W. (2005). Structure and mechanism of the alkyl hydroperoxidase AhpC, a key element of the *Mycobacterium tuberculosis* defense system against oxidative stress. *J. Biol. Chem.* 280 25735–25742. 10.1074/jbc.M503076200 15886207

[B24] Hoehler T. Gunsalus R. P. McInerney M. J. (2010). “Environmental constraints that limit methanogenesis,” in *Handbook of Hydrocarbon and Lipid Microbiology*, eds McGenity T. van der Meer J. R. de Lorenzo V. (Berlin: Springer), 635–654. 10.1007/978-3-540-77587-4_51

[B25] Holmgren A. (1989). Thioredoxin and glutaredoxin systems. *J. Biol. Chem.* 264 13963–13966. 2668278

[B26] Horvath P. Barrangou R. (2010). CRISPR/Cas, the immune system of bacteria and archaea. *Science* 327 167–170. 10.1126/science.1179555 20056882

[B27] Iversen R. Andersen P. A. Jensen K. S. Winther J. R. Sigurskjold B. W. (2010). Thiol-disulfide exchange between glutaredoxin and glutathione. *Biochemistry* 49 810–820. 10.1021/bi9015956 19968277

[B28] Katayama T. Yoshioka H. Mochimaru H. Meng X.-Y. Muramoto Y. Usami J. (2014). *Methanohalophilus levihalophilus* sp. nov., a slightly halophilic, methylotrophic methanogen isolated from natural gas-bearing deep aquifers, and an emended description of the genus *Methanohalophilus*. *Int. J. Syst. Evol. Microbiol.* 64 2089–2093. 10.1099/ijs.0.063677-0 24670897

[B29] Kearse M. Moir R. Wilson A. Stones-Havas S. Cheung M. Sturrock S. (2012). Geneious basic: an integrated and extendable desktop software platform for the organization and analysis of sequence data. *Bioinformatics* 28 1647–1649. 10.1093/bioinformatics/bts199 22543367 PMC3371832

[B30] Kelley C. A. Poole J. A. Tazaz A. M. Chanton J. P. Bebout B. M. (2012). Substrate limitation for methanogenesis in hypersaline environments. *Astrobiology* 12 89–97. 10.1089/ast.2011.0703 22248383

[B31] Konstantinidis K. T. Tiedje J. M. (2005). Genomic insights that advance the species definition for prokaryotes. *Proc. Natl. Acad. Sci. U.S.A.* 102 2567–2572. 10.1073/pnas.0409727102 15701695 PMC549018

[B32] Kozono D. Ding X. Iwasaki I. Meng X. Kamagata Y. Agre P. (2003). Functional expression and characterization of an archaeal aquaporin. AqpM from *Methanothermobacter marburgensis*. *J. Biol. Chem.* 278 10649–10656. 10.1074/jbc.M212418200 12519768

[B33] La Cono V. Smedile F. Bortoluzzi G. Arcadi E. Maimone G. Messina E. (2011). Unveiling microbial life in new deep-sea hypersaline Lake Thetis. Part I: prokaryotes and environmental settings. *Environ. Microbiol.* 13 2250–2268. 10.1111/j.1462-2920.2011.02478.x 21518212

[B34] Lai M. C. Gunsalus R. P. (1992). Glycine betaine and potassium ion are the major compatible solutes in the extremely halophilic methanogen *Methanohalophilus* strain Z7302. *J. Bacteriol.* 174 7474–7477. 10.1128/jb.174.22.7474-7477.1992 1429470 PMC207447

[B35] Lai S.-J. Lai M.-C. (2011). Characterization and regulation of the osmolyte betaine synthesizing enzymes GSMT and SDMT from halophilic methanogen *Methanohalophilus portucalensis*. *PLoS One* 6:e25090. 10.1371/journal.pone.0025090 21949863 PMC3176816

[B36] Lange S. J. Alkhnbashi O. S. Rose D. Will S. Backofen R. (2013). CRISPRmap: an automated classification of repeat conservation in prokaryotic adaptive immune systems. *Nucleic Acids Res.* 41 8034–8044. 10.1093/nar/gkt606 23863837 PMC3783184

[B37] Lazar C. S. Parkes R. J. Cragg B. A. L’Haridon S. Toffin L. (2011). Methanogenic diversity and activity in hypersaline sediments of the centre of the Napoli mud volcano, Eastern Mediterranean Sea. *Environ. Microbiol.* 13 2078–2091. 10.1111/j.1462-2920.2011.02425.x 21382146

[B38] L’Haridon S. Chalopin M. Colombo D. Toffin L. (2014). *Methanococcoides vulcani* sp. nov., a marine methylotrophic methanogen that uses betaine, choline and N,N-dimethylethanolamine for methanogenesis, isolated from a mud volcano, and emended description of the genus *Methanococcoides*. *Int. J. Syst. Evol. Microbiol.* 64 1978–1983. 10.1099/ijs.0.058289-0 24614846

[B39] L’Haridon S. Corre E. Guan Y. Vinu M. La Cono V. Yakimov M. (2017). Complete genome sequence of methanohalophilus halophilus DSM 3094T, isolated from a cyanobacterial mat and bottom deposits at Hamelin Pool, Shark Bay, Northwestern Australia. *Genome. Announc.* 5:e1604-16. 10.1128/genomeA.01604-16 28209822 PMC5313614

[B40] L’Haridon S. Corre E. Guan Y. Vinu M. La Cono V. Yakimov M. (2018). Complete genome sequence of the halophilic methylotrophic methanogen archaeon *Methanohalophilus portucalensis* strain FDF-1T. *Genome Announc.* 6:431. 10.1128/genomeA.01482-17 29348351 PMC5773736

[B41] Lovley D. R. Dwyer D. F. Klug M. J. (1982). Kinetic analysis of competition between sulfate reducers and methanogens for hydrogen in sediments. *Appl. Environ. Microbiol.* 43 1373–1379. 16346033 10.1128/aem.43.6.1373-1379.1982 PMC244242

[B42] Lu J. Holmgren A. (2014). The thioredoxin antioxidant system. *Free Radic. Biol. Med.* 66 75–87. 10.1016/j.freeradbiomed.2013.07.036 23899494

[B43] Magnusson U. Salopek-Sondi B. Luck L. A. Mowbray S. L. (2004). X-ray structures of the leucine-binding protein illustrate conformational changes and the basis of ligand specificity. *J. Biol. Chem.* 279 8747–8752. 10.1074/jbc.M311890200 14672931

[B44] Mahlert F. Bauer C. Jaun B. Thauer R. K. Duin E. C. (2002). The nickel enzyme methyl-coenzyme M reductase from methanogenic archaea: in vitro induction of the nickel-based MCR-ox EPR signals from MCR-red2. *J. Biol. Inorgan. Chem.* 7 500–513. 10.1007/s00775-001-0325-z 11941508

[B45] Makarova K. S. Haft D. H. Barrangou R. Brouns S. J. J. Charpentier E. Horvath P. (2011). Evolution and classification of the CRISPR–Cas systems. *Nat. Rev. Microbiol.* 9 467–477. 10.1038/nrmicro2577 21552286 PMC3380444

[B46] McCarver A. C. Lessner F. H. Soroeta J. M. Lessner D. J. (2017). *Methanosarcina acetivorans* utilizes a single NADPH-dependent thioredoxin system and contains additional thioredoxin homologues with distinct functions. *Microbiology* 163 62–74. 10.1099/mic.0.000406 27902413 PMC5903248

[B47] McGenity T. J. (2010). “Methanogens and methanogenesis in hypersaline environments,” in *Handbook of Hydrocarbon and Lipid Microbiology*, eds McGenity T. van der Meer J. R. de Lorenzo V. (Berlin: Springer), 665–680. 10.1007/978-3-540-77587-4_53

[B48] Ngugi D. K. Blom J. Alam I. Rashid M. Ba-Alawi W. Zhang G. (2015). Comparative genomics reveals adaptations of a halotolerant thaumarchaeon in the interfaces of brine pools in the Red Sea. *ISME J.* 9 396–411. 10.1038/ismej.2014.137 25105904 PMC4303633

[B49] Obraztsova A. Y. Bezrukova V. Belyaev S. S. Shipin O. V. (1987). Properties of the coccoid methylotrophic methanogen, *Methanococcoides euhalobius* sp. *Mikrobiologiia* 56 523–527.

[B50] Oremland R. S. Polcin S. (1982). Methanogenesis and sulfate reduction: competitive and noncompetitive substrates in estuarine sediments. *Appl. Environ. Microbiol.* 44 1270–1276. 16346144 10.1128/aem.44.6.1270-1276.1982 PMC242184

[B51] Oren A. (2011). Thermodynamic limits to microbial life at high salt concentrations. *Environ. Microbiol.* 13 1908–1923. 10.1111/j.1462-2920.2010.02365.x 21054738

[B52] Parks D. H. Imelfort M. Skennerton C. T. Hugenholtz P. Tyson G. W. (2015). CheckM: assessing the quality of microbial genomes recovered from isolates, single cells, and metagenomes. *Genome Res.* 25 1043–1055. 10.1101/gr.186072.114 25977477 PMC4484387

[B53] Paterek J. R. Smith P. H. (1988). *Methanohalophilus mahii* gen nov., sp. nov., a methylotrophic halophilic methanogen. *Int. J. Syst. Bacteriol.* 38 122–123. 10.1099/00207713-38-1-122 11540079

[B54] Prakash D. Walters K. A. Martinie R. J. McCarver A. C. Kumar A. K. Lessner D. J. (2018). Toward a mechanistic and physiological understanding of a ferredoxin:disulfide reductase from the domains archaea and bacteria. *J. Biol. Chem.* 293 9198–9209. 10.1074/jbc.RA118.002473 29720404 PMC6005431

[B55] Sakai S. Takaki Y. Shimamura S. Sekine M. Tajima T. Kosugi H. (2011). Genome sequence of a mesophilic hydrogenotrophic methanogen *Methanocella paludicola*, the first cultivated representative of the order Methanocellales. *PLoS One* 6:e22898. 10.1371/journal.pone.0022898 21829548 PMC3146512

[B56] Schlegel K. Müller D. V. (2011). “Osmoadaptation in methanogenic archaea: physiology, genetics, and regulation in methanosarcina mazei gö1,” in *Extremophiles Handbook*, eds Horikoshi K. Antranikian G. Bull A. T. Robb F. T. Stetter K. O. (Tokyo: Springer), 327–342.

[B57] Seaver L. C. Imlay J. A. (2001). Alkyl hydroperoxide reductase is the primary scavenger of endogenous hydrogen peroxide in *Escherichia coli*. *J. Bacteriol.* 183 7173–7181. 10.1128/JB.183.24.7173-7181.2001 11717276 PMC95566

[B58] Simpson P. G. Whitman W. B. (1993). “Anabolic pathways in methanogens,” in *Methanogenesis: Chapman & Hall Microbiology Series (Physiology / Ecology / Molecular Biology / Biotechnology)*, ed. Ferry J. G. (Boston, MA: Springer). 10.1007/978-1-4615-2391-8_11

[B59] Sorokin D. Y. Makarova K. S. Abbas B. Ferrer M. Golyshin P. N. Galinski E. A. (2017). Discovery of extremely halophilic, methyl-reducing euryarchaea provides insights into the evolutionary origin of methanogenesis. *Nat. Microbiol.* 2:17081. 10.1038/nmicrobiol.2017.81 28555626 PMC5494993

[B60] Sowers K. R. Baron S. F. Ferry J. G. (1984). Methanosarcina acetivorans sp. nov., an acetotrophic methane-producing bacterium isolated from marine sediments. *Appl. Environ. Microbiol.* 47 971–978. 16346552 10.1128/aem.47.5.971-978.1984 PMC240030

[B61] Sowers K. R. Ferry J. G. (1983). Isolation and characterization of a methylotrophic marine methanogen, *Methanococcoides methylutens* gen. nov., sp. nov. *Appl. Environ. Microbiol.* 45 684–690. 16346215 10.1128/aem.45.2.684-690.1983 PMC242344

[B62] Sowers K. R. Gunsalus R. P. (1995). Halotolerance in *Methanosarcina* spp.: role of N(sup(epsilon))-acetyl-(beta)-lysine, (alpha)-glutamate, glycine betaine, and K+ as compatible solutes for osmotic adaptation. *Appl. Environ. Microbiol.* 61 4382–4388. 16535193 10.1128/aem.61.12.4382-4388.1995 PMC1388658

[B63] Spring S. Scheuner C. Lapidus A. Lucas S. Glavina Del Rio T. Tice H. (2010). The genome sequence of *Methanohalophilus Mahii* SLPT reveals differences in the energy metabolism among members of the *Methanosarcinaceae* inhabiting freshwater and saline environments. *Archaea* 2010:690737. 10.1155/2010/690737 21234345 PMC3017947

[B64] Susanti D. Wong J. H. Vensel W. H. Loganathan U. DeSantis R. Schmitz R. A. (2014). Thioredoxin targets fundamental processes in a methane-producing archaeon, *Methanocaldococcus jannaschii*. *Proc. Natl. Acad. Sci. U.S.A.* 111 2608–2613. 10.1073/pnas.1324240111 24505058 PMC3932849

[B65] Sztukowska M. Bugno M. Potempa J. Travis J. Kurtz D. M. (2002). Role of rubrerythrin in the oxidative stress response of *Porphyromonas gingivalis*. *Mol. Microbiol.* 44 479–488. 10.1046/j.1365-2958.2002.02892.x 11972784

[B66] van Der Ploeg J. R. Eichhorn E. Leisinger T. (2001). Sulfonate-sulfur metabolism and its regulation in *Escherichia coli*. *Arch. Microbiol.* 176 1–8. 10.1007/s002030100298 11479697

[B67] Van Laer K. Hamilton C. J. Messens J. (2013). Low-molecular-weight thiols in thiol-disulfide exchange. *Antioxid. Redox Signal.* 18 1642–1653. 10.1089/ars.2012.4964 23075082

[B68] Wilharm T. Zhilina T. N. Hummel P. (1991). DNA-DNA hybridization of methylotrophic halophilic methanogenic bacteria and transfer of *Methanococcus halophilus* vp to the genus *Methanohalophilus* as *Methanohalophilus halophilus* comb. nov. *Int. J. Syst. Bacteriol.* 41 558–562. 10.1099/00207713-41-4-558

[B69] Wolfe R. S. (2011). “Techniques for cultivating methanogens,” in *Methods in Enzymology*, ed. Abelson J. (Amsterdam: Elsevier), 1–22. 10.1016/B978-0-12-385112-3.00001-9 21402207

[B70] Wu M. Scott A. J. (2012). Phylogenomic analysis of bacterial and archaeal sequences with AMPHORA2. *Bioinformatics* 28 1033–1034. 10.1093/bioinformatics/bts079 22332237

[B71] Yakimov M. M. La Cono V. La Spada G. Bortoluzzi G. Messina E. Smedile F. (2014). Microbial community of the deep-sea brine Lake Kryos seawater-brine interface is active below the chaotropicity limit of life as revealed by recovery of mRNA. *Environ. Microbiol.* 17 364–382. 10.1111/1462-2920.12587 25622758

[B72] Yakimov M. M. La Cono V. Slepak V. Z. La Spada G. Arcadi E. Messina E. (2013). Microbial life in the Lake Medee, the largest deep-sea salt-saturated formation. *Sci. Rep.* 3:3554. 10.1038/srep03554 24352146 PMC3867751

[B73] Yelton A. P. Thomas B. C. Simmons S. L. Wilmes P. Zemla A. Thelen M. P. (2011). A semi-quantitative, synteny-based method to improve functional predictions for hypothetical and poorly annotated bacterial and archaeal genes. *PLoS. Comp. Biol.* 7:e1002230. 10.1371/journal.pcbi.1002230 22028637 PMC3197636

[B74] Yenugudhati D. Prakash D. Kumar A. K. Kumar R. S. S. Yennawar N. H. Yennawar H. P. (2015). Structural and biochemical characterizations of Methanoredoxin from *Methanosarcina acetivorans*, a glutaredoxin-like enzyme with coenzyme M-dependent protein disulfide reductase activity. *Biochemistry* 55 313–321. 10.1021/acs.biochem.5b00823 26684934

[B75] Zhilina T. N. (1983). A new obligate halophilic methane-producing bacterium. *Mikrobiologiia* 52 375–383. 3939840

